# Impact of puberty, sex determinants and chronic inflammation on cardiovascular risk in young people

**DOI:** 10.3389/fcvm.2023.1191119

**Published:** 2023-06-27

**Authors:** Amal Allalou, Junjie Peng, George A. Robinson, Crystal Marruganti, Francesco D’Aiuto, Gary Butler, Elizabeth C. Jury, Coziana Ciurtin

**Affiliations:** ^1^ University College London Medical School, University College London, London, United Kingdom; ^2^ Centre for Adolescent Rheumatology Versus Arthritis, University College London, London, United Kingdom; ^3^ Centre for Rheumatology Research, Division of Medicine, University College London, London, United Kingdom; ^4^ Eastman Dental Hospital, University College London Hospital, London, United Kingdom; ^5^ Department of Paediatric Endocrinology, University College London Hospital, London, United Kingdom; ^6^ Institute of Child Health, University College London, London, United Kingdom

**Keywords:** cardiovascular risk factors, children and young poeple, chronic inflammation, puberty, obesity, sex determinants

## Abstract

Worrying trends of increased cardiovascular disease (CVD) risk in children, adolescents and young people in the Modern Era have channelled research and public health strategies to tackle this growing epidemic. However, there are still controversies related to the dynamic of the impact of sex, age and puberty on this risk and on cardiovascular health outcomes later in life. In this comprehensive review of current literature, we examine the relationship between puberty, sex determinants and various traditional CVD-risk factors, as well as subclinical atherosclerosis in young people in general population. In addition, we evaluate the role of chronic inflammation, sex hormone therapy and health-risk behaviours on augmenting traditional CVD-risk factors and health outcomes, ultimately aiming to determine whether tailored management strategies for this age group are justified.

## Introduction

Cardiovascular disease (CVD) is the most common cause of death worldwide (1). Atherosclerosis is one of the earliest signs of CVD. It is characterised by progressive accumulation of cholesterol-laden macrophages in the subendothelial layers of larger arteries which progress to more complex fibrous plaques. Acute plaque rupture or erosion can result in the formation of a thrombus, culminating in the clinical manifestation of myocardial infarction or ischaemic stroke (2). Although more prevalent with increased age, atherosclerotic vascular changes have been documented in young people in multiple post-mortem studies at the end of the past century (3–6). Most notably, asymptomatic microscopic lesions have been described in the coronary arteries of infants in the first 5 years of life, which are thought to precede the more invasive fatty streak lesions associated with atherosclerosis (4, 5). Future studies, built on these findings, established associations between the extent of arterial fatty streaks and fibrotic lesions in young people and traditional CVD-risk factors, such as increasing age and body mass index (BMI), hypertension (HP), dyslipidaemia, aberrant glucose tolerance and smoking, as well as chronic inflammation (3, 7, 8). The presence of CVD-risk factors between the ages of 18–30 can strongly predict the development of subclinical atherosclerosis in later adulthood (9). Interestingly, the sexual dimorphism characterising the CVD prevalence in adulthood is also observed in the prevalence of subclinical atherosclerotic lesions in young people, which were detected in 2% vs. 0% of young men vs. women aged 15–19 years, respectively, and 20% vs. 8% in men vs. women aged 30–34 years, respectively (10). One of the main drivers of this sex difference in prevalence is the higher incidence of traditional CVD risk factors in young males compared to females (6). This is reflected in the sex-disaggregated rate of progression of carotid intima-media thickness (CIMT), which is used as a validated marker of subclinical atherosclerosis, where CIMT progression begins in boys around age 6, and in girls around age 9, and increases gradually with age (11).

Adolescence is a key period of profound physiological changes with significant impact for preservation or deterioration of cardiovascular health, often associated with abnormal cardiovascular health metrics, or significant behavioural changes which impact on diet and other health-related outcomes (12). In addition, sex hormones and genes present on sex chromosomes differentially influence the regulation of the immune system (13), which is also reflected in the sex-bias observed in the predisposition to various autoimmune disorders as well as differential risk for chronic inflammation, which subsequently increases the risk for CVD.

**Figure 1:**
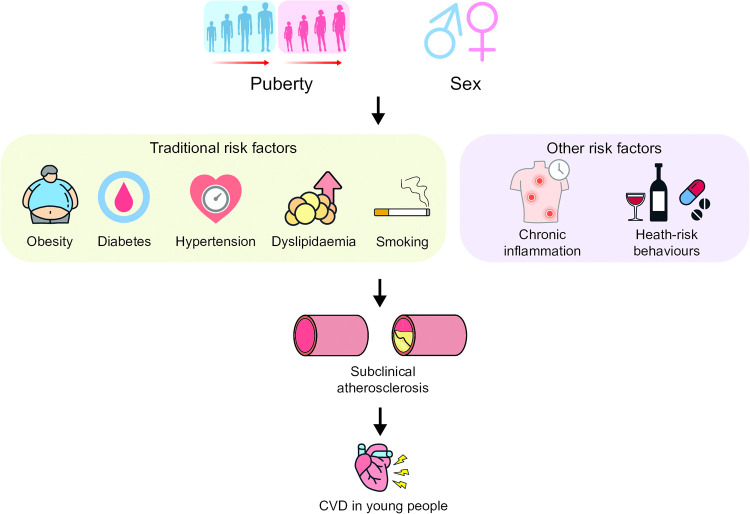
CVD-risk factors yin young populations. Legend: CVD—cardiovascular disease.

In this review, we aim to assess the impact of sex determinants and puberty in driving metabolic abnormalities leading to atherosclerosis progression in young people and CVD-risk later in life, as well as the impact of chronic inflammation and health-risk on augmenting this risk. Identifying CVD-risk factors earlier in life and tailoring management strategies for prevention of CVD is likely to have significant societal implications. We summarised in Table 1 and Figure 1, the main factors contributing to CVD-risk in young people as well as the main effects of puberty and sex determinants which will be discussed in detail in this review.

**Table 1:** Main effects of puberty and sex determinants on traditional CVD-risk factors and subclinical atherosclerosis in young populations.

Risk factors	Impact of puberty	Sex differences
Traditional CVD-risk factors		
Obesity	Increases with puberty.	Both sexes, but stronger effect in girls
	Causes early and shorter puberty.	Stronger effect in girls
	Early puberty is associated with increased obesity risk.	Both sexes, but stronger effect in girls
	In all puberty stages, obesity correlates with increased BP.	Stronger effect in boys
	Increased subcutaneous fat post-menarche.	In girls
	Visceral fat distribution post-puberty.	Stronger effect in boys
	Obesity related biomarkers:	
	IL-6 decreased during puberty.	Both sexes
	Adiponectin decreased during puberty.	Stronger effect in boys
	Leptin increased during puberty	Stronger effect in girls
Metabolic syndrome	Puberty is associated with instability in metabolic syndrome parameters.	In both sexes
	Puberty and adolescents are associated with increased metabolic risk.	Stronger effect in boys
	Adulthood is associated with increased metabolic risk.	Stronger effect in women
Insulin resistance/diabetes	Tanner stages 1–3 increase the risk	Independent of sex or obesity
	Risk increases at the onset of puberty, peaking at Tanner stage 3, but returns to pre-pubertal levels by the end of puberty.	Stronger effect in girls
	Early menarche is associated with increased insulin levels and increased risk of type 2 diabetes mellitus.	In girls
	Obesity increases the risk of insulin resistance post-puberty.	Stronger effect in boys
Dyslipidaemia	Prepuberty, no correlations with atherosclerosis.	In both sexes
	Puberty onset altered lipid profiles	In both sexes
	Post-puberty, there is an increase in pro-atherogenic lipid profile	In young men
	Post-puberty, there is an increase in athero-protective lipid profile	In young women
	After puberty blockers, cross-sex hormone therapy with testosterone drove a pro-atherogenic lipid profile	In young trans-men
Hypertension	Systolic BP increases in children entering puberty.	In both sexes
	BP reaches adult values at the end of puberty	In both sexes
	Puberty timing associated with transitory changes in the BP trajectories.	In both sexes
	In young adulthood, significant increased prevalence of systolic HP which started prepuberty.	In boys and young men
	Endogenous oestrogen exposure starting from puberty decreases the risk	In girls/women
	Risk increases with age, from post-puberty into early adulthood	In boys and young men
Subclinical atherosclerosis		
Carotid artery subclinical atherosclerosis	Puberty increases the prevalence of subclinical atherosclerosis.	In both sexes
Coronary artery atherosclerotic lesions	From age 6–30 (including puberty)	More prevalent in boys/young men
Increased progression of atherosclerosis	Associated with low testosterone and positively correlated with serum oestrogen levels (post-puberty/early adulthood)	In men

### Sex-biased trends in cardiovascular risk factors in young people in general population

Various traditional CVD-risk factors have been studied in young people and have been included in clinical scores/tools aiming at assessing an individual's risk and tailor public health interventions to minimise the risk for CVD over time. We will focus on the main traditional CVD-risk factors from the perspective of the impact of sex-determinants and puberty on their prevalence, features and trends over time.

## Obesity

Between 1975 and 2016, the global prevalence of obesity has nearly tripled (14). As of 2021, WHO estimates an annual mortality of 2.8 million people across all age groups as a direct result of the ongoing obesity epidemic. Targeting excessive weight during childhood and adolescence has been identified as the key intervention in reducing obesity-associated CVD- risk later in life. A higher BMI percentile and central adiposity correlate to a higher risk of obesity and metabolic syndrome in adolescence and adulthood (15, 16). The association between obesity in youth and later life was found to increase with age and is stronger in females (17).

The mechanism by which obesity causes increased CVD- risk is multifactorial. Obesity is associated with chronic mild inflammation, largely mediated by the secretion of inflammatory adipokines by excessive adipose tissue. Higher adiposity throughout puberty was associated with a more atherogenic metabolic profile and greater aortic stiffness (independent of metabolic factors), while reverting to a healthy weight during adolescence prevents this association and results in no difference in arterial stiffness compared to a normal weight control cohort (18). Not all obese individuals are at increased CVD-risk. A high body fat percentage in both adults and children (without additional features of metabolic syndrome—i.e., high blood glucose, insulin resistance, dyslipidaemia) is considered a metabolically healthy obese (MHO) state, and is not associated with increased CVD-risk (19, 20). However, children with prepubertal obesity are at a greater risk of metabolic syndrome, and the pronounced changes to their metabolic parameters during puberty often cause the shift to a metabolically unhealthy status (21, 22).

We summarise below the main effects of obesity on CVD-risk in young populations by sex, including its timing and its bidirectional relationship with puberty.

*Obesity in childhood increases the risk of obesity in adulthood and CVD-risk factors in a sex biased way* (15). A higher BMI percentile during childhood and adolescence correlates with a high risk of being overweight or obese in adulthood, and the association becomes stronger as age increases. The link observed is stronger in females than in males (17). During all stages of puberty, there is a positive link between trunk fat and systolic and diastolic blood pressure (BP). This association is seen in males only (23).

*Obesity is associated with a pro-inflammatory state from an early age*. Obesity associated with chronic mild inflammation led to elevated inflammatory markers, including C-reactive protein (CRP), Tumour Necrosis Factor-α (TNF-α), interleukin (IL)-6, IL-18, haptoglobin, macrophage inhibitory factor (MIF) and plasminogen activator inhibitor-1 (24). White adipose tissue has been noted to play an endocrine role, secreting leptin and adiponectin (25, 26). Obesity (particularly visceral fat) in childhood and adolescence increased the risk of metabolic syndrome in adulthood and adolescence, independent of baseline insulin level (16), suggesting that lifestyle factors may contribute more to the development of metabolic syndrome in young people than hereditary factors (27).

*Obesity affects the timing of puberty*. Obesity causes early and shorter puberty in girls (28–30), which can be explained by the “weight hypothesis”, which suggests that adipokines increase androgen conversion to oestrogen, therefore influencing the timing of puberty. Earlier puberty in obese children is determined by an interplay between molecular factors such as leptin, insulin and oestrogen. There is evidence that serum leptin levels are inversely correlated with age at menarche in girls (31, 32), while there was a close positive correlation between body fat and serum leptin levels in girls throughout puberty (31–35). Children with obesity have lower sex hormone-binding globulin than normal weight children (36), while obese adolescent boys have lower serum testosterone and higher oestradiol than normal weight adolescent males (36, 37). Overweight girls have reduced sleep-associated luteinising hormone (LH) synthesis, the first hormonal change observed at the onset of puberty (38).*Puberty timing affects the obesity risk.*. Early puberty in girls may be related to obesity due to the effect of oestradiol in increasing the body fat, while early menarche was associated with increased CVD-risk and mortality (39, 40). Early menarche was also related to reduced sex hormone binding globulin and higher oestrogen levels throughout adolescence and into adulthood (41–43), as well as increased risk of metabolic syndrome in adulthood, independent of BMI (44–50)*.* Early puberty is associated with higher accumulation of subcutaneous fat on lower trunk in both males and females (49). The large AVENA study (50) assessed more than 500 children and adolescents and found that the waist circumference and BMI increased in girls as puberty continued, while in boys there were no correlations with the pubertal stage. An increase in total body fat was observed in both females and males throughout puberty, and BMI increased through the 8–18 years interval of observation (51). This was largely driven by the increase in the lean component of BMI, particularly in boys (52). Pubertal stage also affects the adipokine profile (53), and inflammation caused by obesity was somewhat affected by pubertal changes in sex hormones (54).

*Obesity related biomarkers are also influenced by puberty and sex hormones*. Although no significant difference between prepubertal and pubertal levels of leptin were found independent of body fat content (35), circulating IL-6 levels correlated positively with testosterone and oestradiol levels, and leptin: receptor ratio correlated positively with BMI in both sexes. In addition, IL-6 decreased throughout puberty in both boys and girls but was only found to be correlated to oestradiol and not testosterone. Adiponectin decreases in males from mid-puberty to levels below the ones found in females (53) and levels of circulating androgens were found to decrease plasma adiponectin and may be the cause for increased risk of insulin resistance and atherosclerosis in men (55). Higher serum leptin levels were observed in girls than males pre-, during and post-puberty, even after correcting for increased female adiposity. It is proposed that this may be due to oestradiol stimulating leptin synthesis and testosterone suppressing it (56). At the onset of puberty, leptin increases in girls and decreases in boys, potentially reflecting the increase in fat mass in girls (34), in addition to the potential role of testosterone in suppressing the leptin production (57). By the age of 15, there is an increase in IL-6, IL-8, IL-10 serum concentrations in obese and overweight girls compared to normal weight females even after adjustment for pubertal status, but no significant difference was observed in boys (58).

However, the relationship between early and shorter puberty on CVD-risk later in life is less certain. Data from the Avon Longitudinal Study of Parents and Children (ALSPAC) which recruited children born between April 1, 1991, and December 31, 1992 who were followed-up for 25 years, concluded that earlier puberty was unlikely to have a major impact on pre-clinical CVD-risk in early adulthood, appreciated using a variety of CVD validated outcome measures (59).

*There are significant sex differences in adipose tissue distribution around puberty*. Over the course of puberty, the prevalence of obesity or excess weight status (measured by BMI) doubles in girls (60). Post menarche, girls have greater subcutaneous fat deposits than pre-menarcheal girls, particularly in the gluteo-femoral region, therefore the sexual dimorphism in fat distribution begins in or is triggered by early puberty (61, 62). Higher oestrogen correlated with gynoid fat distribution in pubertal females (63). Age at menarche negatively correlated to hip and thigh circumference and negatively correlated to waist circumference (61), while early menarche associated with increased adiposity in childhood and increased risk for metabolic syndrome in adulthood (64). In boys, higher serum testosterone was associated with increase in subcutaneous abdominal fat deposition during puberty (63).

*Sexual dimorphism in storage of adipose tissue (fat distribution) may underlie the increased CVD-risk in men*. Visceral fat and fat deposition around abdomen are favoured in men (central android fat distribution), while subcutaneous fatty deposition in women is largely observed around hips, thighs, and buttocks (gynoid/ gluteal-femoral fat distribution) until menopause, after which there is a shift towards increased deposition of visceral fat (65, 66). Lipoprotein lipase is one of the key enzymes that facilitates the accumulation of adipose tissue. Testosterone inhibits lipoprotein lipase activity in subcutaneous fat in men (67). Lipoprotein activity in women is greater in gluteal subcutaneous fat than in abdominal visceral fat, and higher in abdominal fat in men (68). Distribution of receptors that modulate lipolysis in subcutaneous and intraabdominal fat depots account in part for the sexual dimorphism in body fat deposition. Oestradiol upregulates expression of anti-lipolytic *α*2-adrenergic receptors in subcutaneous adipocytes but not in abdominal depots (69). The ratio of *α*2 receptors to lipolytic *β*1–2 adrenergic receptors is higher in subcutaneous adipose tissue in young women than in men, promoting lower rates of lipolysis in these depots and a reduced lipolytic response to adrenaline and noradrenaline (70).

Increased visceral fat is associated with decreased insulin resistance, increased risk of glucose intolerance and metabolic syndrome (71, 72). Visceral fats have a higher lipolytic rate (65). Higher rate of lipolysis of visceral fat, as observed in males, releases more free fatty acids into the circulation, which in turn causes increased gluconeogenesis and hyperinsulinaemia (73). Subcutaneous fat on the other hand is associated with a lower CVD-risk and do not have the same diabetes-associated risk (74).

Despite good evidence that puberty and sex determinants influence obesity in young people, there is evidence of obesity risks that precede puberty. Children with one obese parent are more than twice as likely to be obese (75), and increased maternal pre-pregnancy weight and gestational weight gain increases the child's adverse CVD-risk. Children of mothers with higher gestation weight gain had higher BMI, waist circumference, fat mass, leptin, CRP and IL6 levels, as well as high density lipoprotein (HDL)-cholesterol and Apolipoprotein (Apo) A1) (76).

In conclusion, obesity in childhood is associated with increased traditional CVD-risk factors overall, as well as increased risk of obesity in adulthood. Obesity triggers early and shorter puberty, while early puberty also predisposes to obesity. There is also evidence of sex dimorphism in fat distribution and obesity-related biomarkers underpinning sex-differencs in CVD-risk from adolescence and young adulthood.

## Metabolic syndrome

Metabolic syndrome is defined as a combination of risk factors for CVD, such as diabetes, HP, dyslipidaemia and obesity. The diagnosis of metabolic syndrome in children and adolescents is slightly controversial as only 50% of adolescents diagnosed with metabolic syndrome maintained a stable diagnosis over 3 years (77) and only 30% of obese children and adolescents diagnosed with metabolic syndrome at baseline were found to still maintain the required characteristics at a 60-day follow up (78). This instability may be a result of the dynamic changes observed throughout puberty. Metabolic syndrome in adolescence is higher in males than females by ∼10% (79), but this trend reverses with age as it becomes more prevalent in adult women (80). There is no correlation between any circulating metabolites and measures of atherosclerosis in children, but an inverse correlation between HDL-cholesterol levels and measures of atherosclerosis independent of BMI and BP are present in adulthood (81). However, CVD-risk is nine times higher in children with metabolic syndrome (82). Children with one parent with metabolic syndrome were also at greater risk of obesity and insulin resistance (75). In conclusion, puberty may promote metabolic instability, with a reversal of the sex-bias from male to female from adolescence into adulthood.

**Insulin resistance** is measured as impaired fasting glucose or impaired glucose tolerance. Hyperglycaemia measured indirectly through levels of glycohemoglobin in post-mortem studies was found to be associated with increases fatty streaks and raised coronary atherosclerotic lesions in young people who died in road traffic accidents from the age of 25, and aortic lesions from the age of 30 (83). In adolescence, obese males have a higher risk for insulin resistance and impaired fasting glucose than obese females (84). Insulin resistance increases at the onset of puberty, peaking at Tanner stage 3, but returns to baseline pre-pubertal levels by the end of puberty (85) and girls are more insulin resistant than boys at all pubertal stages. Serum insulin is highest at Tanner stage 2 in both sexes, independent of body mass and retains a consistent level throughout puberty (86). Between Tanner stages 1–3, there is an increase in fasting glucose, insulin and acute insulin response and an associated reduction in insulin sensitivity independent of sex or obesity status. The reduction in insulin sensitivity was not associated with androgen, oestradiol, visceral fat, or IGF-1 (87) and high levels of insulin in childhood were associated with early menarche (64).

Earlier menarche associated with increased risk of type 2 diabetes mellitus (T2DM) in adulthood independent of BMI at 18 years of age (45). This implies a potential role for sex hormones in the pathophysiology of T2DM (46). Hyperandrogenic profiles in women (e.g., polycystic ovary syndrome) are associated with insulin resistance and glucose intolerance (47, 48), while high oestradiol, high testosterone and low sex hormone binding globulin associated with increased risk of T2DM in women, independent of BMI (46, 88, 89).

In conclusion, puberty onset and early timing of puberty, as well as hyperandrogenic profiles in women increase the insulin resistance and diabetes risk, while early phases of puberty are associated with increased risk in both sexes, independent of obesity.

### Dyslipidaemia

Prevalence of dyslipidaemia in children and adolescents across the world is increasing, being four-times more common in children and adolescents with obesity (90). The relationship between lipid abnormalities and atherosclerosis or vascular dysfunction in young people is currently debated (81) as no correlation between lipids and CIMT or pulse wave velocity has been found in children aged 11–12, independent of their BMI or BP. However, associations between these vascular markers and HDL-cholesterol and glucose levels were found in adulthood, suggesting that perhaps these associations arise during/post- puberty. Changes in lipid profiles that occur in puberty however are relevant, as they predispose to increased CVD-risk in adulthood (91). Overall, higher concentrations of triglycerides, low density lipoproteins (LDL) and very low-density lipoproteins (VLDL) cholesterol and ApoB were found in men, with corresponding higher concentrations of HDL-cholesterol, ApoA1, polyunsaturated fatty acids (PUFA) and docosahexaenoic acid (DHA) in women (92, 93). Interestingly, cross-sex hormone therapy in young trans-sexual individuals undergoing gender-reassigning treatment partially reversed the sex bias observed post-puberty in healthy controls (93).

#### Sex differences in dyslipidaemia

Although no sex difference has been observed in the mean total cholesterol concentration in the Framingham Offspring Study (92), there were sex differences in HDL, LDL and VLDL particles size, which are potentially influenced by sex hormones. Oestrogens have a role in promoting an atheroprotective lipid profile. High serum oestrogen promoted VLDL and LDL clearance as well as increased synthesis of HDL in mouse models through mediation of the hepatic oestrogen receptor *α* (Er*α*), the actions of which have been proposed to be modulated by an interaction with Liver X receptor *α* (LXR*α*) (94). Contrastingly, cellular study analysing HDL efflux in macrophages in trans-gender women undergoing hormone therapy found reduction in HDL efflux, with a specific decrease in ATP-binding cassette transporter A1 mediated HDL efflux (95).

No sex difference between total HDL concentrations have been reported in the large Framingham Offspring Study either (92). However, young men had a greater concentration of small and intermediate HDL particle subclasses, while women had almost double the concentration of large HDL particles (92). Although women have larger HDL particles than men, this difference is less prominent with increasing age (92). There is some evidence for a stronger inverse correlation between CVD-risk and HDL-cholesterol levels in women than men (96), and this could be attributable to the size of HDL particles (92). This trend seems to be related to the presence of female sex hormones as increased oestradiol serum concentrations correlated with increased HDL-cholesterol in young trans women too (93). ApoA1 is a metabolite for HDL cholesterol which is also affected by sex bias. Women have higher HDL-associated ApoA1 than men while ApoA1 levels were positively corelated with the length of oestradiol therapy and oestradiol serum concentrations in young trans women on cross-sex hormone therapy. However, there was no correlation with testosterone levels in young trans men (93).

Men have a greater total LDL-cholesterol serum concentration than women (92) and smaller LDL particles than women (92, 97–99). This is likely due to a greater concentration of small and intermediate LDL particle subclasses in men, and a greater concentration of large LDL subclasses in women (92). It has been suggested that the sex difference in total LDL particle concentration may be responsible for the sex bias observed in CVD-risk, as the sex difference in LDL concentrations also decreases with age, similar to the trend in CVD risk. There is an increase in small LDL in men before 50 that might underlie the increased CVD risk in middle aged men compared to younger men (92).

Men also have a higher VLDL-cholesterol serum concentration (92), and in particular this affects the larger and intermediate VLDL subclasses as men overall have larger mean VLDL particle sizes (92).

#### Puberty affects the lipid profiles

 Although, no significant difference in lipid profiles were detected between prepubertal girls and boys (93), the onset of puberty altered lipid profiles (100), although these changes normalised post-puberty, raising awareness that changes observed during this puberty should be considered in clinical guidelines for CVD-risk-assessment in adolescents and young people (101).

In conclusion, post-puberty, there is evidence for a trend towards a pro-atherogenic lipid profile in young men or trans-men undergoing cross-sex hormone gender re-assignment therapy with testosterone, while young women have higher concentrations of athero-protective HDL-associated ApoA1.

### Hypertension (HP)

It is widely recognised that arterial BP increases with age (102), although has been also gradually increasing in prevalence in younger populations in the recent years Primary HP has been found to be more prevalent in children over age of 6, with associated increased BMI and positive family history (103). The most common causes for secondary HP in children and adolescents are congenital malformations, renal diseases, medications (such as corticosteroids, albuterol and pseudoephedrine), and endocrine causes, while in adolescents, additional factors are related to substance abuse and teen pregnancy (104).

#### Impact of puberty on HP

Despite less research available overall in younger populations compared to adults, there is evidence that the hormonal and metabolic changes associated with puberty impact BP. The overall prevalence of confirmed HP in children is 2%–5%, with many more being diagnosed with pre-hypertensive states (15.3%) (105). While initial research identified low birth weight as a risk factor for HP in childhood (106, 107), more recent studies found evidence that infants born with appropriate for gestational age or excessive birth weight are both at higher risk for primary HP compared to children with small weight for gestational age (odds ratio = 1.31 and 1.19 respectively) (107). Systolic BP increases in children entering puberty, reaching adult values at the end of puberty, process considered to be significantly influenced by the increased production of sex hormones, growth hormone, insulin-like growth factor-1 and insulin during puberty (108).

Puberty timing also influences the risk of HP. In a large prospective cohort study, which evaluated more than 4,000 children over time, males had significantly increased prevalence of systolic HP compared with females in early adulthood, but this was accrued before puberty, while puberty timing was associated only with small transient differences in systolic BP trajectories post-puberty in both sexes, suggesting that interventions targeting puberty timing are unlikely to influence systolic BP in early adulthood (109–114).

#### Sex differences in HP are underpinned by various mechanisms

The inhibition of the renin-angiotensin-aldosterone system (RAAS) is a recognised therapeutic option for HP; however, there is evidence that suggests responses to treatment is different in men and in women (115). Endogenous oestrogen is associated with a lower BP in women (116), while there is no consensus from literature data on the effect of exogenous oestrogen, which has been found to decrease (117–119), increase (120–122) or not affect BP measurements (123, 124). These differences may have arisen due to the administration of different oestrogenic compounds at different doses through different routes of administration, and different methods used to measure BP (125). The protective effect of female sex hormones could be explained by the role of oestrogen in promoting vasodilation and exerting cardioprotective effects on the RAAS system. In animal models, oestrogen has been found to upregulate synthesis of angiotensinogen and downregulate synthesis of renin and angiotensin enzyme (ACE) (124, 126, 127), leading protective effects. In rats, oestrogen has been found to reduce calcium efflux in vascular, renal and cardiac cells (128) as well as reduce BP by inhibiting synthesis of angiotensin II (AT II), endothelin 1 (ET1) and catecholamines that all have vasoconstrictive effects (124, 129, 130). One of the mechanisms of oestrogen's effect on BP is mediated through its modulation of expression of ET1, which is a potent vasoconstrictor and its receptors (131). The administration of exogenous 17*β*-oestradiol to ovariectomised rats decreased the circulating ET1 levels (132, 133) and reduced the expression and activity of endothelin-converting enzyme (133, 134). In vitro studies also find that 17*β*-oestradiol inhibits ET1 synthesis by increasing nitric oxide synthase (135) +/- decreasing AT II synthesis (136, 137).

Testosterone is pro-hypertensive, through potential modulation of the plasma ET1 levels, resulting in ET1 levels that are 40% higher in men and 90% higher in trans men treated with testosterone therapy than in premenopausal women (138, 139). Testosterone also promotes vasoconstriction and renal sodium reabsorption through the stimulation of AT1 receptor (AT1R) (140–142). In animal studies, it has been noted that males have a higher vascular AT1R:AT2R ratio than females (127, 143, 144).

#### Sex differences in Hp prevalence in adolescents and young adults

Sex differences in HP in adulthood are well-recognised, with males being more at risk; however, sex differences are particularly pronounced in early adulthood, with one study reporting both sex and ethnic differences in HP among 18- to 29-year-old adults (1.5% in White women vs. 5% White men and 4% and 10% for Black women and men, respectively) (145).

In a large American study of a large representative sample of adolescents followed up to age 24–34, young women were far less likely to be hypertensive compared to men (12% vs. 27%), and there were also sex differences in HP awareness among young people (32% and 25% of hypertensive women and men, respectively were aware of their status) (146).

In conclusion, the prevalence of HP is increased overall from the onset of puberty in both sexes, reaching adult values at the end of puberty, and is significantly influenced by molecular mechanisms subjected to sex hormones and metabolic factors regulation driving the overall increased risk in young males.

##### Subclinical atherosclerosis in young people in general population

As a consequence of various CVD-risk factors encountered in young population, there is also evidence of presence of subclinical atherosclerosis, usually evaluated on vascular scans or post-mortem studies. Subclinical atherosclerosis is one of the best predictors of CVD later in life. Coronary artery atherosclerotic lesions measured post-mortem in individuals aged 6–30 were more prevalent in men than women (6) All the traditional CVD-risk factors influence the prevalence of subclinical atherosclerosis. Puberty was associated with increased subclinical atherosclerosis in children with a benign obesity phenotype (147). In young people, HP contributes to atherosclerosis by accelerating development of raised lesions rather than fatty streaks. A post-mortem study in people aged 15–34 found that HP, causing increased intimal thickness of small renal arteries, was associated with more extensive raised atherosclerotic lesions in the abdominal aorta and in the right coronary artery (148). A post-mortem histological study of coronary arteries from American young people aged 15–34 found a higher prevalence of advanced atherosclerotic lesions in young men compared to young women (10). Positive correlation between serum oestrogen and progression of carotid atherosclerosis was found in men with intact oestradiol synthesis independent of BMI and other risk factors (149), while low testosterone in men associated with increased progression of atherosclerosis (measured by CIMT) (149).

In conclusion, there is a significant male sex bias in prevalence of subclinical atherosclerosis from early childhood into adulthood.

##### Effect of chronic inflammation on the cardiovascular risk of young people

Chronic inflammation is one of the known drivers of CVD-risk in patients of all ages. In addition to inflammation, some of the treatments that are used in young people with chronic inflammatory conditions (in particular, corticosteroids or small molecule immunosuppressants, such as Janus Kinase inhibitors and biologic treatments, such as IL6 blockers, etc) have significant metabolic effects (150), which indirectly impact the CVD-risk of a certain individual. Assessment of disease activity in young people with chronic inflammation usually pertain to composite scores aiming to combine objective and subjective assessment of disease manifestations, as well as laboratory parameters which are relevant for systemic inflammation or immune system abnormalities contributing to chronic inflammation (151–153). Despite improvement in quantifying inflammation and improved guidelines to ensure tighter control of disease activity in young people with chronic inflammatory and autoimmune conditions (154–156), there is still evidence of increased CVD-risk in children and young people which is not adequately captured by the CVD-risk assessment tools routinely used for primary care prevention strategies in general population(157, 158).

Long-term inflammation as well as glucocorticoid treatment used in the treatment of many childhood onset diseases, such as juvenile idiopathic arthritis (JIA), juvenile systemic lupus erythematosus (JSLE), juvenile dermatomyositis (JDM), inflammatory bowel disease (IBD), etc, influence the body composition by increasing body fat mass and reducing skeletal muscle mass (159). Obesity is more common in patients with JIA than in the healthy population (160, 161) and is associated with insulin resistance, HP, higher serum triglycerides and early atherosclerosis (162). Obesity and excess weight in JIA population is caused, in part, by glucocorticoid treatment, and functional limitation that causes a less active lifestyle (163) Children with systemic JIA and enthesitis-related arthritis (ERA) had the highest rates of overweight and obesity compared to other JIA subtypes (163). Several studies explored the association between obesity and disease activity or number of active and reported both no association (164, 165), and a positive correlation between obesity and disease activity (particularly on lower limb joints), number of affected joints and higher levels of CRP and ESR compared to the healthy population (166). Children with JIA and a BMI lower than 23 kg/m^2^ had lower serum leptin than healthy subjects, while children who were overweight or obese had higher prevalence of insulin resistance, lower insulin sensitivity and higher insulin secretion than age matched overweight or obese healthy children. In lean children with systemic JIA, insulin sensitivity was not different to lean age-matched controls (167).

In JDM, a condition associated with muscle and skin inflammation, a unique adipokine profile was found, characterised by higher serum adiponectin and resistin, and lower leptin levels in young women (168). A study of 17 patients with severe JDM, found a very high prevalence of obesity and excess weight, as well as insulin resistance and hypertriglyceridaemia (169). The increased CVD-risk observed in JDM is very likely multifactorial, largely accounted for by chronic inflammation, steroid treatment and poor functional levels leading to increased weight in some patients (170).

No significant difference in the prevalence of obesity in children and adolescents with JSLE compared to healthy controls has been reported (171), although there was a positive correlation between obesity (BMI) in JSLE patients and higher serum levels of TNF-α than in healthy controls. TNF-α is a pro-inflammatory adipokine, associated with a decreased activity of lipoprotein lipase in adipose tissue. It also has a role in the early inflammatory response that contributes to atherosclerosis. Furthermore, it is associated with hyperglycaemia, insulin resistance and dyslipidaemia, mediated by inhibition of insulin receptor autophosphorylation and signal transduction (172, 173). In a small study, it has been found that the prevalence of metabolic syndrome in patients with JSLE was higher than in the healthy population (174).

Children and adolescents with T1DM also had more severe periodontal inflammation (175), which was also associated with increased BMI in young people (176). Periodontal disease is also associated with atherosclerosis risk, potentially from early in life (177). In addition, chronic conditions, such as type II DM (T2DM), RA and SLE which are recognised risk factors for CVD are also associated with periodontal disease, although the majority of studies are derived from adult populations, characterised by a higher prevalence of gum disease (178–180).

Higher prevalence of dyslipidaemia was also observed in young people with T1DM characterised by increased triglycerides and LDL-cholesterol levels, with female adolescents having lower levels of HDL-cholesterol than healthy controls (181). Children with T1DM have higher triglyceride levels, independent of pubertal stage (unlike in healthy controls where TC decreased throughout puberty) which also correlated strongly with ApoB levels (182).

#### Impact of sex determinants on chronic inflammation and other cardiovascular risk factors relevant to young people's health

There is a recognised female sex bias in autoimmune rheumatic diseases associated with chronic inflammation, although this is less pronounced in diseases with paediatric onset (183). This sex-bias is due to a combination of genetic and epigenetic mechanisms (184), as well as due to impact of sex determinants at the time of puberty on the disease mechanisms and risk overall (185, 186). Many of the autoimmune conditions in young people are rare diseases overall. Therefore, it is not always easy to tease apart sex differences in CVD-risk due to chronic inflammation as many cohort studies are underpowered to detect this. Although, this sex bias is less evident in T1DM (187) and has inverse trends in IBD, where there is evidence of females have a lower risk of Crohn's disease compared with males until puberty, at which point there is a reversal, with females developing higher risk over time (188), the long-term impact of increased CVD-risk in patients with chronic inflammation is less defined by sex differences, suggesting that chronic inflammation is the main driver of this risk (189). However, there is evidence that younger age is an independent factor from increased CVD-risk in patients with RA, which is one of the most prevalent and better studied inflammatory rheumatic conditions (190), suggesting that chronic inflammation rather than increase in prevalence of traditional CVD-risk factor are likely to impact cardiovascular health overall.

No post-pubertal sex differences in serum lipid levels were found between adult men and women with JSLE (93), although in female adolescents with JSLE, dyslipidaemia was more than two times more prevalent than in healthy controls, and these differences were characterised by a lower serum HDL and a higher homocysteine in JSLE cohort (191). Dyslipidaemia in JSLE was also associated with decreased smaller HDL particle subsets than in healthy controls. Active disease accentuated this difference and was further associated with higher VLDL particles when compared to JSLE patients with lower disease activity, and it was related to B- and T-cell lipid rafts, inflammation, and disturbed liver function (192). JSLE patients with active disease also had increase in ApoB:ApoA1 ratio which is a pro-atherosclerotic biomarker (192). In addition, JSLE patients with a high ApoB:ApoA1 ratio (baseline levels correlated positively to SLE disease activity index) had increased cardiometabolic risk conferred by greater number of CD8+ T-lymphocytes and CD8+ T-lymphocyte transcriptomic profile which expressed a higher number of genes associated with interferon signalling and other processes that contribute to atherosclerosis (193).

In conclusion, while chronic inflammatory conditions in adolescents and young people are associated with an increased CVD-risk overall, this is likely driven by both traditional and non-traditional CVD-risk factors, and it is less influenced by puberty or other sex-determinants as the effect of underlying chronic inflammatory disease and treatment seem to override their impact.

##### Other cardiovascular risk factors relevant for young people

 

## Role of (cross-) sex hormones, hormonal contraception, and hormone replacement therapy (HRT) on cardiovascular risk

The role of sex hormones in the development of atherosclerosis has been a point of contention. It has been suggested that sex-hormones have differential effects in either sex, although there are less published data in young people (194).

Low endogenous testosterone has been linked to increased CVD and mortality in men, suggesting an atheroprotective role in men, albeit through an unclear mechanism (195). It has also been argued however, that causation is uncertain, and low testosterone may be reflective of reduced general health status in men and thus indicative of other factors that may be directly linked to CVD-risk rather than the causative factor itself (196).

There is evidence of increased CVD-risk with oestrogen-combined oral contraceptives in women of reproductive age, in particularly the risk of venous thrombosis (2–7-fold), as well as arterial thrombosis and HP (197, 198). As a consequence, despite benefits in preventing unwanted pregnancies, as well as addressing other medical conditions common in adolescent and young females, such as menstrual irregularities, heavy menstrual bleeding, menstrual discomfort, or required to treat endometriosis, polycystic ovary syndrome, acne and ovarian cysts, combined oral contraceptives are not recommended (WHO-MEC 3) or even contraindicated (WHO-MEC4) in women with cardiac disease, increased thrombotic risk, either venous or arterial, ischaemic heart disease or HP (199).

Emergency contraception (Levonogestrel) has a small effect on blood clotting parameters and increases fibrinogen at 24 and 48 h post-dose, while being associated with a reduction in anti-thrombin III lasting from 2 to 12 h post treatment (200). However, despite these changes in laboratory parameters, there was no evidence of an increased risk of thrombosis in users of emergency contraception (201).

Large-scale studies thus far have been unable to establish a benefit of HRT for cardioprotective purposes. There is however some evidence for a benefit if treatment started immediately post-menopause, leading to the “timing hypothesis”, which suggests that the type of oestrogen used and the time between the start of menopause and initiation of HRT can lead to differential cardiovascular outcomes (202). However, no data are available in younger populations as this as sex hormone therapy is very rarely warranted. The impact of cross-sex hormone therapy in young trans-sex populations is insufficiently studied (203) as there are unmet needs to involve gender-diverse people in research in medicine overall (204).

In conclusion, while associated with a small CVD-risk overall, sex hormone therapies containing oestrogen have to be used cautiously in young female populations at risk, especially as alternatives are available (progesterone only contraceptive preparations or intrauterine devices and barrier protection).

## Sex differences in health-risk behaviours contributing to cardiovascular risk in young people

According to the WHO, 10% of mortality due to CVD can be attributed to cigarette smoking (205) Smoking was associated with more extensive fatty streaks and raised atherosclerotic lesions in the abdominal aorta of young people in post-mortem study (206). Inhalation of cigarette smoke, both passively and actively, increases CVD-risk, as 10% of smoking-related mortality can be attributed to passive cigarette smoking (207). Smoking increases the risk of myocardial infarction and stroke (208) and exposure to cigarette smoke affects the regulation of mechanisms responsible for the formation of intravascular thrombi, inducing a hypercoagulable state and contributing to increased risked of acute thrombotic events (208). There is a higher mortality rate in female smokers than male smokers, with female smokers being 25% more likely to develop coronary heart disease (209).

A study conducted on healthy young men and women found that CVD-risk factors occur earlier in young female smokers than male cigarette smokers (210). In women only, exposure to cigarette smoke increased monocyte and lymphocyte counts, whereas in men, neutrophils and eosinophils were significantly increased. Global DNA methylation was reduced more in women than men who smoked, while smoking increased the number of platelets in women, and decreased the number of platelets in men. Smoking also seemed to affect endothelial function in women more than in men by causing a significant increase in asymmetric dimethyl arginine (ADMA), an endogenous inhibitor of nitric oxide synthesis, and L-arginine, a precursor of nitric oxide synthesis in women only. These are both surrogate measures of endothelial dysfunction. The trans-sulphuration pathway involves interconversion of cysteine and homocysteine. The forward reaction in this pathway produces homocysteine, a recognised marker of CVD-risk (211). In the non-smoking control group, homocysteine was found to be lower in females than in males, while exposure to cigarette smoke increased homocysteine levels in women only. Smoking also eradicated the sexual dimorphism in TNF-α release from human monocyte-derived macrophages (hMDMs) as even if in non-smokers, there is a higher basal TNF-α release from hMDMs in men than in women, the smoking increased TNF-α release in women only. Early onset of puberty associated with increased risk of smoking throughout adolescence (212).

Cigarette smoking intensity (acute smoking measured as number of cigarettes smoked per day) has been associated with increased serum concentration of biomarkers of CVD, particularly, markers of systemic inflammation, with hsCRP, being the most sensitive (213).

A large meta-analysis found a dose-dependent relationship between the number of cigarettes smoked per day, with pooled relative risk for coronary heart disease in men of 1.48 for smoking one cigarette per day and 2.04 for 20 cigarettes, while in women, the risk was 1.57 and 2.84, respectively (214).

Long-term exposure (chronic cigarette smoking), commonly assessed using smoking duration (years), or cumulative exposure (pack-years) was also associated with measures of either inflammation or subclinical atherosclerosis (215), which is relevant to adolescents, especially as cigarette smoking initiation rates during early adolescence (11–15 years) showed a marked increase after 1990 especially in West Europe, while smoking initiation during late adolescence (16–20 years) declined, with the exception of South Europe (216).

Recent research has been directed towards understanding the cardiovascular health effects of electronic vaping cigarettes (EVC) and heat-not-burn cigarettes, which are popular alternatives to traditional combustion cigarettes. An analysis of 7 systematic reviews found that acute EVC use was associated with several toxic effects, including detrimental impact on BP management, tachycardia, worsened arterial stiffness, as well as increased prevalence of atrial fibrillation and myocardial infarction, even if the causal link is still debated (217).

Adolescence is also associated with increased use of illicit substances (218), with potential devastating impact on cardiovascular health (219). Many studies investigated sex and gender differences as well as contextual factors and relationships associated with substance use and academic and health-related outcomes (220–224), highlighting the complexity of this phenomenon which cannot be simply disaggregated by sex. Significant atherosclerosis has been particularly linked to cocaine use compared to opioid use and other poor-health related factors (225). Illicit substance use is one of the main drivers of CVD in young people overall (226).

### Is there any need for tailored CVD-risk strategies in young people?

There is ample evidence in the literature that puberty and adolescence are very dynamic periods in the life of young people associated with various metabolic changes and CVD-risk trends, some of which are reversable. Correct phenotyping of an individual, as well as assessment of risk over time are important strategies for managing the potential health consequences of having increased CVD-risk or subclinical atherosclerosis later in life. We would argue that certain groups of young people, especially in the context of genetic predisposition, concomitant chronic inflammatory conditions or at the time of intense vulnerability driven by physiological, socio-economic or psychological factors would benefit from tailored management strategies, irrespective of being aimed at addressing chronic inflammation, improving physical exercise or diet, or tackling health-risk associated behaviours. In addition, early identification of increased CVD-risk through improved detection strategies as well as dynamic assessment of this risk over time are likely to lead to improved outcomes. Although, there is evidence of a male bias in increased CVD-risk in young people, especially post-puberty, future research is needed to establish whether this can be addressed by sex-biased therapeutic and management strategies from early life.

## Conclusions

There is an unmet need for better CVD-risk assessment and management strategies in young people overall, as although the traditional risk factors are clearly linked to subclinical atherosclerosis, there are other contributing determinants, related to pubertal changes, chronic inflammation and treatment addressing inflammation, as well as health-risk behaviours that are particularly relevant in this population. There are well documented sex differences in CVD-risk and subclinical atherosclerosis which maintain the male-biased predominance observed in the older populations; however, in the context of chronic inflammatory conditions, the upregulation of the pro-inflammatory pathways or the use of various treatments associated with metabolic effects with role in CVD-risk modulation seem to override the sex and puberty driven differences observed in the general population. Further research is needed to capture the long-term outcomes of young people with chronic inflammatory diseases and contrast them with the impact of traditional CVD-risk factors in the general population, disaggregated by sex, to give us the possibility to properly investigate whether sex and gender-tailored CVD-risk management strategies are warranted.
